# Efficacy, Immunogenicity and Safety of Vaccination in Pediatric Patients With Autoimmune Inflammatory Rheumatic Diseases (pedAIIRD): A Systematic Literature Review for the 2021 Update of the EULAR/PRES Recommendations

**DOI:** 10.3389/fped.2022.910026

**Published:** 2022-07-06

**Authors:** Marc H. Jansen, Christien Rondaan, Geertje Legger, Kirsten Minden, Yosef Uziel, Nataša Toplak, Despoina Maritsi, Mirjam van den Berg, Guy Berbers, Patricia Bruijning, Yona Egert, Christophe Normand, Marc Bijl, Helen Foster, Isabelle Kone-Paut, Carine Wouters, Angelo Ravelli, Ori Elkayam, Nicolaas M. Wulffraat, Marloes W. Heijstek

**Affiliations:** ^1^Department of Paediatric Immunology & Rheumatology, Wilhelmina Children's Hospital, University Medical Centre Utrecht, Utrecht, Netherlands; ^2^RITA, European Reference Networks, Brussels, Belgium; ^3^Department of Medical Microbiology and Infection Prevention, University Medical Centre Groningen, University of Groningen, Groningen, Netherlands; ^4^Department of Paediatric Rheumatology, Beatrix Children's Hospital, University Medical Centre Groningen, University of Groningen, Groningen, Netherlands; ^5^Department of Paediatric Respiratory Medicine, Immunology and Critical Care Medicine, Deutsches Rheuma-Forschungszentrum Berlin, Charité - Universitätsmedizin Berlin, Corporate Member of Freie Universität Berlin and Humboldt- Universität zu Berlin, Berlin, Germany; ^6^Epidemiology Unit, German Rheumatism Research Centre, Berlin, Germany; ^7^Paediatric Rheumatology Unit, Department of Paediatrics, Meir Medical Centre, Sackler School of Medicine, Tel-Aviv University, Tel-Aviv, Israel; ^8^Department of Allergology, Rheumatology and Clinical Immunology, University Children's Hospital Ljubljana, Ljubljana, Slovenia; ^9^Infectious Diseases, Immunology and Rheumatology Unit, Second Department of Paediatrics, Medical School, P. & A. Kyriakou Children's Hospital, National and Kapodistrian University of Athens, Athens, Greece; ^10^Dutch JIA patients and Parent Organisation (JVN), Amsterdam, Netherlands; ^11^Centre for Infectious Disease Control, Laboratory for Infectious Diseases and Screening, National Institute for Public Health and the Environment (RIVM), Bilthoven, Netherlands; ^12^Julius Center for Health Sciences and Primary Care, University Medical Center Utrecht, Utrecht, Netherlands; ^13^European Network for Children With Arthritis (ENCA), MCI Secretariat, Geneva, Switzerland; ^14^Department of Rheumatology and Clinical Immunology, Martini Hospital Groningen, Groningen, Netherlands; ^15^Population and Health Institute, Newcastle University, Newcastle upon Tyne, United Kingdom; ^16^Department of Paediatric Rheumatology and CEREMAIA, Bicêtre University Hospital, Université Paris Saclay, Paris, France; ^17^Division of Paediatric Rheumatology University Hospitals Leuven, Leuven, Belgium; ^18^Direzione Scientifica, IRCCS Istituto Giannina Gaslini, Dipartimento di Neuroscienze, Riabilitazione, Oftalmologia, Genetica e Scienze Materno-Infantili (DINOGMI), Università degli Studi di Genova, Genoa, Italy; ^19^Department of Rheumatology, Sackler Faculty of Medicine, Tel Aviv Sourasky Medical Center, Tel Aviv University, Tel Aviv, Israel; ^20^Department of Rheumatology & Clinical Immunology, University Medical Centre Utrecht, Utrecht, Netherlands

**Keywords:** vaccination, pediatric rheumatic disease, pediatric immunology, immunosuppressants, vaccination responses, safety

## Abstract

**Background:**

In 2011, the first European League Against Rheumatism (EULAR) vaccination recommendations for pediatric patients with autoimmune inflammatory rheumatic diseases (pedAIIRD) were published. The past decade numerous new studies were performed to assess the safety, efficacy and immunogenicity of vaccinations in pedAIIRD. A systematic literature review (SLR) was therefore performed to serve as the basis for the updated 2021 EULAR/PRES recommendations.

**Methods:**

An SLR was performed according to the standard operating procedures for EULAR-endorsed recommendations. Primary outcomes were efficacy, immunogenicity and safety of vaccination in pedAIIRD. The search was performed in Medline, Embase and the Cochrane Library and included studies published from November 2010 until July 2020.

**Results:**

The SLR yielded 57 studies which were included for critical appraisal and data extraction. Only 8 studies described the occurrence of vaccine-preventable infections after vaccination (efficacy), none of these studies were powered to assess efficacy. The majority of studies assessed (humoral) immune responses as surrogate endpoint for vaccine efficacy. Studies on non-live vaccines showed that these were safe and in general immunogenic. Biologic disease-modifying antirheumatic drugs (bDMARDs) in general did not significantly reduce seroprotection rates, except for B-cell depleting therapies which severely hampered humoral responses. Four new studies on human papilloma virus vaccination showed that this vaccine was safe and immunogenic in pedAIIRD. Regarding live-attenuated vaccinations, level 1 evidence of the measles mumps rubella (MMR) booster vaccination became available which showed the safety of this booster for patients treated with methotrexate. In addition, level 3 evidence became available that suggested that the MMR and varicella zoster virus (VZV) vaccination for patients on low dose glucocorticosteroids and bDMARDs might be safe as well.

**Conclusions:**

The past decade, knowledge on the safety and immunogenicity of (live-attenuated) vaccines in pedAIIRD significantly increased. Data on efficacy (infection prevention) remains scarce. The results from this SLR are the basis for the updated EULAR/PRES vaccination recommendations in pedAIIRD.

## Introduction

Vaccination is one of the greatest interventions that has been established to reduce mortality rates in children (1). Patients with autoimmune and/or inflammatory rheumatic diseases (AIIRD) have an increased risk of infection both due to their disease and more importantly to the use of immunosuppressive medication (2–4). For these patients, it is therefore even more important to prevent severe viral and bacterial infections. However, due to their immunosuppressed status, the safety of especially live-attenuated vaccines and the capacity of vaccines to induce protective immune responses is a matter of concern.

In 2011, The European League Against Rheumatism (EULAR) presented the recommendations on vaccination of pediatric patients with autoimmune/inflammatory rheumatic diseases (pedAIIRD) (5). Very few studies were available on pedAIIRD patients and data from studies performed in adult patients had to be extrapolated. Since 2011, numerous trails have been published that studied both live-attenuated and non-live vaccines and that assessed the effects of biologic disease modifying antirheumatic drugs (bDMARDS) on the outcomes of vaccination in specifically pedAIIRD.

We therefore performed a systematic literature review (SLR) on the safety, immunogenicity, and efficacy of vaccinations in pedAIIRD, to serve as the basis for the updated 2021 EULAR vaccination recommendations for pedAIIRD.

## Methods

### Systematic Literature Review

The SLR was performed according to the 2014 EULAR standard operating procedures for EULAR-endorsed recommendations (6). The original SLR performed in 2011 served as a starting point (5).

The research question was: what is the safety, efficacy or immunogenicity of vaccines in pedAIIRD, including patients treated with immunosuppressive agents? (Supplementary File 1). Safety was defined as occurrence of (severe) adverse events, effect of vaccination on underlying disease and whether live-attenuated vaccine induced infections; efficacy was defined as the capacity of vaccines to prevent vaccine-preventable infections (VPIs); immunogenicity was defined as the ability to induce humoral and cellular immune responses after vaccination. The immunogenicity of vaccines is often used as a surrogate primary endpoint for efficacy. This is a valid method when there is good correlation between pathogen-specific antibody levels and protection against infection such as for measles, rubella, hepatitis A and B. For other VPIs this correlation is less clear, for example HPV (7). This will be indicated throughout the paper.

MJ, MH and CR were in charge of the SLR. The research question was adapted into search terms according to the PICO method (patient-intervention-comparison-outcome). All available vaccines were included in the search, except for the COVID vaccine. Terminology for medication is according to the new nomenclature of DMARDs, including conventional synthetic (cs)DMARDs, targeted synthetic (ts)DMARDs and biological (b)DMARDs (8). Search terms were combined and are shown in the Supplementary File 1.

Medline (via Pubmed) and Embase were searched for literature published between November 2010 and July 2020. The list was further extended by reviewing the reference lists of identified papers to check for studies that might have been missed in the search strategy. All original studies, including case-reports were eligible for inclusion.

**Figure 1:**
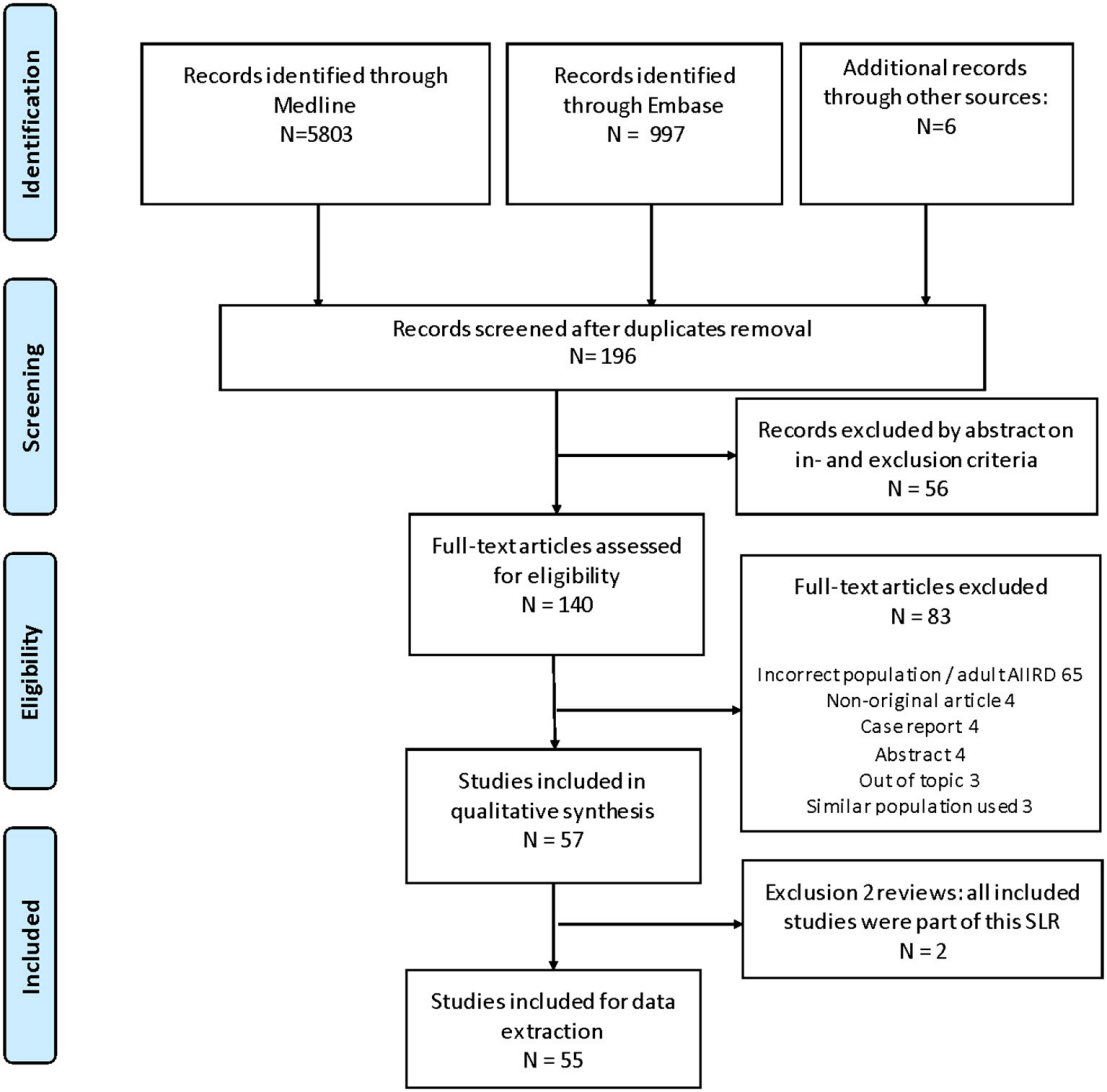
Prisma flow chart of SLR Nov 2011–July 2020.

Exclusion criteria were studies that focussed exclusively on non-rheumatic autoimmune diseases [except for inflammatory bowel disease (IBD)] or vaccine development. Phase I trials, *in vitro* studies, non-English papers and abstracts presented on scientific meetings were also excluded. Papers concerning the potential role of vaccinations in *inducing* pedAIIRD were excluded, because these recommendations focus on the effect of vaccination on *established* disease. The flow chart of the search is depicted in Figure 1.

Data analysis was performed by MH, MJ and NW. Data on study design, number and type of pedAIIRD, control group, medication use and the three outcomes (safety, efficacy, immunogenicity) were extracted. The quality of the studies was critically assessed using standardized critical appraisal criteria and levels of evidence (LoE) were determined using the standards of the Oxford Centre for Evidence-Based Medicine (Supplementary Tables 1, 2). Each paper was evaluated by at least two experts. The steering committee organized a 1-day meeting of the Task Force. Prior to this meeting, all experts read and independently graded literature on methodological quality and level of evidence. Results and discrepancies were discussed, followed by the formulation and grading of the recommendations.

The final manuscript was drafted after the meeting, reviewed, revised and approved by all Task Force members, followed by final review and approval by the EULAR Executive Committee before submission to the journal.

## Results

The SLR yielded 57 studies that were included for data extraction. The studies included 2 systematic reviews, 2 randomized controlled trials (RCTs), 52 cohort studies and 1 case report. The two systematic reviews were eventually excluded from the analysis as all included studies were part of this review. Some studies investigated multiple vaccines (Table 1). The studies covered the non-live vaccines against Diphtheria Tetanus Polio (DTP, 6 studies), Hepatitis A virus (HAV, 4 studies), Hepatitis B virus (HBV, 7 studies), Human Papillomavirus (HPV, 4 studies), Influenza (13 studies), Meningococcus C (1 study) and Pneumococci (both the pneumococcal conjugate vaccination (PCV) and/or the 23-valent pneumococcal polysaccharide vaccine (PPSV-23) vaccine, 6 studies) (9–50). In addition, studies were included that reported on the live-attenuated vaccines against Measles, Mumps and Rubella (MMR, 12 studies, including 3 on the combined MMR-Varicella booster) (12–14, 51–59), Varicella Zoster Virus (VZV, 5 studies) (59–63), one study which included 1 patient with oral polio vaccine (59) and one case report on the Bacillus Calmette-Guérin (BCG) vaccine (64).

**Table 1:** Critical appraisal of the literature on the efficacy, immunogenicity and safety of vaccinations in pedAIIRD.

									**LoE**		
**References**	**Vaccine**	**Design**	**Patients (No)**	**Medication**	**Efficacy**	**Immunogenicity**	**Safety (AE)**	**Safety (Disease activity)**	**Eff**	**Imm**	**Saf**
**DPT**											
Banaszkiewicz et al. (9)	Pertussis	Cohort	138: 109 IBD 29 HC	55 Thiopur vs. 20 Thiopur + TNFi	NA	No difference in titers 8 wks post vacc between therapies; IS vs. no IS & pts vs. HC	Local reactions. No systemic AE	No flares	NA	2b	2b
Dembiński et al. (10)	Diphtheria	Cohort	32 IBD on IS, 14 without IS	32 on IS (6-MP, AZA, GC, MTX, CYC, bDMARD)	NA	No differences IS vs. no IS	8 mild AE, injection related	No flares	NA	2b	4
Brunner et al. (11)	DT	cohort	29 pJIA, no HC	29 ABT, 22 MTX, 3 LD-GC	NA	100% SP tetanus, 90% SP diphtheria 2 m post vacc	SAE 4, AE 29 (all ABT-, no vacc.-related)	NA	NA	4	4
Miyamoto et al. (12)	TT	Cohort	30 jSLE, 14 HC	Unknown	NA	Reduced GMT TT in jSLE vs. HC	NA	NA	NA	2b	NA
Ingelman-Sundberg et al. (13)	TT	Cohort	46 JIA, 1 PAN, 1 MCTD, 1 JDM, 1 other	10 NSAID, 8 MTX, 32 MTX+TNFi, 31 age-m. HC	NA	IgG-TT reduced in MTX+TNFi group.	NA	NA	NA	2b	NA
Heijstek et al. (14)	DT	Cohort	400 JIA, 2176 HC	93 MTX, 8 TNFi, 28 GC 10 mg/day	NA	Reduced SP and GMT for tetanus. No effect MTX or GC	NA	NA	NA	2b	NA
**HPV**											
Heijstek et al. (15)	HPV	Cohort	68 JIA, 55 HC	24 MTX, 9 TNFi, 6 other DMARD	NA	Equal SC and GMT in JIA and HC	AE similar in JIA and HC, no SAE	SD 1 year post vacc.	NA	2b	4
Heijstek (50)	HPV	Cohort	6 jSLE, 6 JDM, 49 HC	6 GC, 1 AZA, 1 MMF, 2 HCQ, 2 MTX, 5 no medication	NA	Equal SC. IgG titers lower (ns for SLE, sign. for JDM at 7 months post-vacc)	NA	1 flare, 2 lower SLEDAI. JDM: SD	NA	3b	NA
Soybilgic et al. (16)	HPV	Cohort	22 jSLE, 8 pts <18 yrs	27 HCQ, 16 GC, 9 MMF, 9 AZA, 6 MTX	NA	SC 94% for all serotypes. No controls. Only 16 samples available	NA	9 mild/mod. SLE flares. 2/12 renal failure (unrel.to HPV)	NA	4	4
Esposito et al. (17)	HPV-b	Cohort	21 JIA, 21 HC	10 NSAID, 5 MTX, 6 TNFi	NA	100% SC. Reduced titers 1 month post 3th vacc in JIA vs. HC. No effect of medication	Similar AE as HC	No increase in JADAS-27	NA	2a	4
**HBV/HAV**											
Moses et al. (18)	HBV	Cross-sectional	100 IBD	100 IFX	NA	49/87 (56%) SP in vacc. pts. No relation with IFX dose, freq. or duration, no controls	NA	NA	NA	4	NA
Maritsi et al. (55)	HAV	Cohort	28 PFAPA, 76 HC	13 NSAID, 9 GC and NSAID, 3 no medication	NA	No difference pts vs. HC, no effect of medication	6 PFAPA & 15 HC: mild fever, malaise	3 flares, No effect medication	NA	2b	2b
Maritsi (19)	HAV	Cohort	21 jSLE 76 HC	GC, HCQ, AZA	NA	Equal SP. jSLE: increased AB loss	NA	NA	NA	2b	NA
Mertoglu et al. (24)	HAV	Cohort	30 jSLE, 39 HC	11 GC, 15 HCQ, AZA, MMF, RTX	NA	Equal SP, anti HAV IgG lower in jSLE	No (S)AE	No change SLEDAI-2K, IQR	NA	2b	4
Maritsi (19)	HAV	Cohort	83 JIA, 76 HC	83 MTX	NA	Reduced SP after 1 vacc, equal after 2. Reduced GMT in pts vs. HC.	No SAE	NA	NA	2b	4
Maritsi et al. (22)	HBV	Cohort	89 JIA, 89 HC	unknown	NA	Reduced SP & GMT in pts vs. HC	NA	NA	NA	3b	NA
Szczygielska et al. (25)	HBV	Cohort	56 JIA	MTX &: 27 ETN, 20 ADA, 9 TCZ	NA	61% SP. No data on effect medication	NA	NA	NA	4	NA
Watts et al. (27)	HBV	cohort	116 IBD: groups: 5–10, 10–15, 16–18yr	15 GC, 66 DMARDs, 53 TNFi	NA	SP 60% in 5-10yrs vs. 22% in 10–15 & 27% in 16–18yrs	NA	NA	NA	4	NA
Nerome et al. (28)	HBV (10 ug)	Cohort	25 JIA	9 GC, 19 MTX, 9 TCZ, 4 ADM, 3 ETN, 2 IFX	NA	Equal SP in biologicals vs. non-biologicals (76 vs. 86%)	no SAE	SD pre & postvacc.	NA	2c	4
Szczygielska et al. (26)	HBV (20 ug)	cohort	50 pedAIIRD (28 JIA, 10 SSc, 5 MCTD, 2 SLE, 5 other), 50 HC	46 GCs, 41 MTX, 8 AZA, 1 MMF, 6 CYC, 3 IVIG, 9 TNFi, 1 other	NA	Reduced SP in pts vs. HC (50 vs. 62%)	NA	NA	NA	2b	NA
Aytac et al. (29)	HBV	Cohort	20 jSLE, 24 HC	17 GC (<15 mg/day), 11 AZA, 3 MMF, 2 HCQ	NA	N.s. reduction in SC (jSLE 80%, HC 100%) & GMT in jSLE; no effect of medication	NA	Flares 15% vs. 18% in “other pts”	NA	2b	NA
**MenC**											
Stoof et al. (30)	MenC	Cohort	127 JIA, 1527 HC	42 MTX, 66 pre-MTX, 7 bDMARD, 53 pre-, 4 GC, 10 pre-GC	NA	Equal SP 4 yrs post vacc JIA vs. HC. MenC-IgG decrease over time, ↑ trend bDMARDs (*n* = 7)	NA	NA	NA	2c	NA
**PCV/PPV**											
Gorelik et al. (31)	PCV	Cohort	26 SLE	26 HCQ, 14 GC, 8 Cy, 13 MMF, 5 AZA, 9 RTX, 1 ABA, 3 MTX	1 pt on RTX got pneumonia	65% SP for PCV13, 59% for PPSV23. RTX within 6 months pre-vacc reduced SP for PCV13	NA	NA	4	4	NA
Banaszkiewicz et al. (32)	PCV13	Cohort	122 IBD, 56 HC	IBD no IS vs IBD HD IS (80 AZA, 2 CYC, 12 TNFi)	NA	Equal SP (90% IBD vs. 97% HC, ns). No difference in GMT between therapies.	Similar EA	NA	NA	2b	2b
Walker et al. (33)	PCV13	Cohort, 1 child	1 pediatric CAPS patient	no medication	NA	NA	Large local reaction requiring topical therapy	No flare	NA	NA	4
Jaeger et al. (34)	PPV23, PCV	Cohort, 18 children	CAPS; 9 influenza, 3 TT, 1 PCV, 1 PPSV23, other 4	All on CAM	NA	NA	Not specified for children vs. adults. In total 0% AE after PCV, AE to PPSV23 were more severe (flares)	Flares	NA	NA	4
Aikawa (35)	PPV23	Cohort	17 JIA (pre-etanercept), 10 JIA MTX	Group 1: MTX HD 2 weeks before ETA. Group 2: 10 LD MTX	Pneumoc. Invasive dis. in pt on TNFi: serotype NA.	Equal SC at 2 (53 vs. 30%) and 12 months (36 vs. 40%) in JIA with and without TNFi	No increased AE. 1 SAE: pneumococcal invasive dis.	SD in JIA pts	4	2b	4
Alyasin et al. (36)	PPV23	Cohort	30 SLE, 30 asthma	24 HCQ, 30 pred, 3 AZA, 5 MTX, 5 Cy, 5 MMF, 4 other	NA	2-fold incr. SC:77,7% SLE, 86,2% HC. Adeq. SP in SLE. Poor resp. had higher SLEDAI scores	Safe	No raise in SLEDAI	NA	2b	4
**Influenza**											
Aikawa et al. (38)	H1N1	Cohort	93 JIA, 99 jSLE, 18 JDM, 11 jScl, 16 vasculitis, 91 HC	90 GC, MTX 74, 43 AZA, 23 CYC, 13 MMF, 6 LEF, 3 Cy	NA	Reduced SC jSLE vs. HC. With GC lower GMT, esp. GC >20 mg/day. Multiv. regr. only GC sign.	Mild AE, similar in pts and HC. More arthralgia in pedRD	NA	NA	2b	4
Aikawa (35)	H1N1	Cohort	95 JIA, 91 HC	16 TNFi, 63 DMARD(s)	NA	Equal SP and GMT. Reduced SC in pts, irresp.of TNF/MTX	Mild AE, similar in pts and HC. More arthralgia in JIA	DAS stable	NA	2b	4
Campos et al. (39)	H1N1	cohort	118 jSLE, 102 HC		NA	Reduced SP, SC & GMT in JSLE. Non-SC assoc. with SLEDAI>8	no SAE	SLEDAI stable	NA	2b	4
Guissa et al. (40)	H1N1	cohort	31 JDM, 81 HC (18 patients from previous study)		NA	Equal SP & GMT	AE similar in JDM and HC	SD	NA	2b	4
Laestadius et al. (41)	Influenza	Cohort	78 PRD, 22HC	17 non-treated, 14 MTX, 36 TNFi	NA	Equal SP 3 m. post-vacc. H1N1b (93–100%), H3N2(37–53%), B(33%)	No SAE	NA	NA	4	4
Carvalho et al. (42)	Influenza	Cohort	44 JIA, 10 HC	IS in 34-44%	Pos. influenza samples 5/14 vs. 1/7 & ILI ↑ in unvacc vs. vacc.	Equal SP & SC in JIA and HC. TNFi lower SP and SC for H1N1 but SP for h3N2 &B/Florida was normal (N = not reported).	NA	Equal ACRped30 pre & post vacc	4	2b	4
deBruyn et al. (43)	Influenza	Cohort	60 IBD, 53 HC	2 GC, 32 DMARDs (AZA or 6-MP or MTX), 8 bDMARDs	NA	Equal SP in IBD vs. HC (95% & 98% to H3N2, H1N1)	1SAE: pancreatitis	1 flare DA scores stable	NA	2b	2b
Shimizu et al. (44)	Influenza	Cohort	1 SJIA	anti-IL6	NA	NA	NA	2x arthritis post-vaccin	NA	NA	4
Shinoki et al. (45)	Influenza	Cohort	27 SJIA	27 TCZ+GC	1 SJIA with influenza.	SP, SC & GMT similar in SoJIA and HC	4 pts with mild AE, none in HC	No flares	4	2b	4
Toplak et al. (46)	Influenza	cohort	31 JIA, 14 HC	18 NSAID, 2 DMARD, 7 DMARD+GC, 4 TNF	Equal influenza JIA vs. HC. Infections in 1 vacc. vs. 4 unvacc. pts	Equal SP pts and HC	Similar AE	Flare rate at 6 mths: 36% vs. 23% vacc vs. control JIA (but lower basel. DA)	4	2b	2b
Woerner et al. (47)	Influenza	cohort	25 JIA, 3 uv, 1 vasc, 1 SLE, 1 MCTD, 2 IBD, 1 JDM, 16 HC	18 MTX, 10 TNFi, 8 MTX+TNFi, 16 no medication	NA	Equal SP pts vs. HC, reduced GMT. In multiv. anal. no effect MTX/TNFi	Similar AE	NA	NA	2b	4
Dell'Era et al. (48)	Influenza (M59 adjuvanted)	cohort	60 JIA, 30 HC	30 DMARD vs. 30 aTNF (Etanercept)	NA	Equal SP & SC in JIA and HC. TNFi lower H1N1-GMT & more rapid decline in H3N2-GMT	AE similar in JIA and HC	Stable DA during follow-up	NA	2b	2b
Camacho-Lovillo et al. (49)	Influenza (H1N1, H3N2, B)	cohort	25 JIA	anti TNF, anti IL-1, anti IL-6	NA	SP after 4–8 wks: 97.8% H1N1, 95.6% H3N2, 91.1% B. No effect medication	No severe AE. 7 /41 local reactions, 2/41 systemic AE: drug reactions	No flares, increased JADAS in 6pt	NA	2b	4
**MMR**											
Heijstek et al. (51)	MMR	Cohort	137 JIA (68 MMR, 69 no vaccin)	60 MTX, 15 biologicals, 3 GC	NA	SP and GMT higher in vacc. Vs. controls, no effect medication	No MMR infections induced by vaccination	Stable DA, incl. pts on biologicals	NA	1b	1b
Tacke et al. (52)	MMR	Cohort	155 kawasaki, 155 HC	92 IVIG before MMR1, 58 IVIG after MMR1, 5 no IVIG	NA	MMR1 pre-IVIG vs. HC: equal respons. MMR1 post-IVIG vs. HC: lower GMT & SP until 9 mths post-IVIG	NA	NA	NA	2c	NA
Kraszewska-Głomba et al. (53)	MMR	Cohort	31 PFAPA, 22 HC	no medication	NA	Equal SP of measles & rubella and reduced SP of mumps (64 vs. 95%) in PFAPA vs. HC 3 yrs post 1th MMR vacc.	NA	NA	NA	3b	NA
Maritsi et al. (54)	MMR focus Measles	Cohort	21 jSLE 76 healthy controls	No information	NA	Adequate SP in pts and HC. Reduced measles-IgG and GMCs in jSLE at 1 & 3 yrs FU	NA	NA	NA	2b	NA
Maritsi 2018	MMR focus Rubella	cohort	21 jSLE 76 healthy controls	21 GC, 21 HCQ, 9 AZA	NA	Adequate SP in pts and HC. Reduced rubella-IgG in jSLE at diagn, 1 & 3 yrs FU.	NA	NA	NA	2b	NA
Heijstek et al. (14)	MMR	Cohort	400 JIA, 2176 HC	93 MTX, 8 TNFi, 28 GC 10 mg/day	NA	Reduced SP and GMT for mumps, rubella, but not measles. No effect of MTX or GC	NA	NA	NA	2b	NA
Ingelman-Sundberg et al. (13)	MMR	Cohort	46 JIA, 1 PAN, 1 MCTD, 1 JDM, 1 other	10 NSAID, 8 MTX, 32 MTX+TNFi, 31 age-m. HC	NA	Equal IgG titres MV and RV, IgG-TT reduced in MTX+iTNF group. Vacc.-spec. mem. B cells preserved in pts with booster	NA	NA	NA	2b	NA
Lee et al. (56)	12MMR(/V), (&28 rota)	Cohort	38 Kawasaki	IFX + IVIG within 90 days after MMR/V	NA	no data	1 MMR-vacc. pt with urticaria after IFX, further no AE	NA	NA	NA	4
Cagol et al. (57)	MMR/V	Cross-sectional	329 IBD, AIH	283 (86%) on IS, both DMARDs, GC & 75 TNFi	NA	99% ≥1 MMR vacc. SP 89% for measles. SP VZV in vacc pts 93/118 (75%) No effect TNFi	NA	NA	NA	2b	NA
Uziel et al. (58)	MMR/V	Cohort	211 JIA (18 sJIA), 1 AID, 1 MKDS, 1 FMF, 5 SCD. 5 uv., 11 JDM	71 MTX & biological, 39 biologics only, 124 MTX only, 5 with <20 mg/day GCS	NA	NA	9 Mild AE. No vacc.-related infection of MMR/V	No flares	NA	NA	4
Jeyaratnam et al. (59)	MMR	Cohort	7 pts (4 sJIA, 1 FMF, 1 CAPS, 1 MKD)	2 CAM, 4 ANR, 1 TCZ (GC, MTX, colc)	NA	NA	1 pneumonia (sJIA on CAM)	1 sJIA flare (also pneumonia)	NA	NA	4
Miyamoto et al. (12)	MV	Cohort	30 jSLE, 14 HC	unknown	NA	Equal MV-in pts vs. HC	NA	NA	NA	2b	NA
**VZV**											
Groot et al. (60)	VZV	Cohort	39 JIA, 5 JDM, 5 jSSC, 18 HC (25 pts also in pileggi et al.)	49 MTX, 16 GCs, 3 biologials	3 low responder pts had VZV inf.	Equal GMT in pts & HC, more VZV-spec. T cells.	3 pts with self-limiting vesicular rash	No change in DA	3	2b	4
Barbosa et al. (61)	VZV booster	RCT	54 jSLE (28 vac, 26 control), 28 HC(all VCV seropositive)	Vaccinated jSLE: 18 GC, 9 AZA, 2 MTX, 27 HCQ	36 m. follow-up: 4 HZ infection	SP pre-vac 100% in jSLE and HC. Similar increase in VZV-GMT.	NA	Flares similar in vacc. Vs. unvacc. jSLE	1b	1b	4
Toplak et al. (62)	VZV booster	Cohort	6 JIA pts on biologicals	3 ETN+MTX, 2 TCZ+MTX+Tacro, 1 IFX+MTX	NA	SP in 83% after booster, but decline in time	No SAE, no VZV infection in 3 mo post-vacc.	SD disease after booster	NA	4	4
Speth et al. (63)	VZV, primo	Cohort	23 PRD VZV seronegative	LD-IS 9 MTX <15 mg/week, 14 HD- IS (including 4 TNFi, 2 TCZ, 2 ANR, 1 ABT, 4 GCs, 5 LEF)	3 yrs post-vacc no VZV infection, 3 pts exposed	No difference in SC between LIIS and HIIS.	minor AE LISS group: No rash or vacc. induced VZV	No flares	2b	2b	4
Jeyaratnam et al. (59)	VZV 2, VZVb 3	Cohort	5 pts VZV (2 sJIA, 3 MKD)	3 ANR, 1 CAM, 1 TCZ (GC, MTX, LEF, thal)	NA	NA	1 varicella zoster infection	3 flares	NA	NA	4
**Oral polio**											
Jeyaratnam et al. (59)	Oral polio	Cohort	1 sJIA	TCZ & pred	NA	NA	Diarrhea (probably vaccine-induced)	No flare	NA	NA	4
**BCG**											
Cheent et al. (64)	BCG	CR	1 newborn	Maternal use of infliximab	NA	NA	Fatal case of disseminated BCG infection	NA	NA	NA	4

*LoE, level of evidence; Eff, Efficacy; Imm, immunogenicity Saf, Safety; NA, not analyzed; SP, seroprotection; SC, seroconversion; GMT, geometric mean titer; HC, healthy controls; pts, patients; DTP, diphtheria tetanus pertussis; IBD, inflammatory bowel disease; Thiopur, thiopurine; TNFi, tumor necrosis factor inhibitor; AE, adverse event; SAE, severe adverse event; IS, immunosuppression; JIA, juvenile idiopathic arthritis; jSLE, juvenile systemic lupus erythematosus; 6-MP, 6-mercaptopurine; AZA, azathioprine; GC, glucocorticosteroids; MTX, methotrexate; CYC, cyclosporine; bDMARD, biological disease modifying anti-rheumatic drugs; ABT, abatacept; LD, low dose; HD, high dose; TT, tetanus toxoid; MV, measles vaccine; NSAID, non steroid anti-inflammatory drug; HCQ, hydroxychloroquine; MMF, mycophenolic acid; RTX, rituximab; vacc, vaccine; AB, antibody; HAV, hepatitis A virus; HBV, hepatitis B virus; ETN, etanercept; ADA, adalimumab; TCZ, tocilizumab; IFX, infliximab; IVIG, intravenous immunoglobulines; Cy, cyclophosphamide; LEF, leflunomide; MMR(/V), measles mumps rubella (/varicella); VZV, varicella zoster virus; ANR, Anakinra; CAM, canakinumab; FMF, familial mediterranean fever; CAPS, cryopyrin-associated periodic syndrome; MKD, mevalonate kinase deficiency*.

### Non-live Attenuated Vaccines

#### Diphtheria, Tetanus, Pertussis

##### Efficacy–Immunogenicity–Safety

Since the 2011 recommendations, six cohort studies including in total 650 pedAIIRD patients and 2219 healthy controls assessed the DTP (3 diphtheria, 1 pertussis, 4 tetanus) vaccination, with a maximum level of evidence of 2B for immunogenicity and safety (adverse events) (9–14). Efficacy was not evaluated; all studies assessed immunogenicity. Twenty-nine patients with polyarticular juvenile idiopathic arthritis (pJIA) on abatacept mounted high levels of seroprotection rates (90–100%), whereas 30 patients with juvenile onset systemic lupus erythematosus (jSLE) showed lower antibody concentrations against tetanus toxoid than 14 healthy controls (11, 12).

With regard to safety, no severe vaccine-related adverse events were described and no disease flares were detected in vaccinated patients.

##### Influence of Treatment

No differences were found in antibody titers or adverse events between IBD patients with various csDMARDS (6-mercaptopurine, azathioprine, methotrexate), glucocorticosteroids and bDMARDS (TNFα-inhibitors) compared to IBD patients without these drugs (9). Also in JIA patients, medication did not influence the seroprotection titers (14).

Based on these data, the national immunisation program (NIP) can be followed for pedAIIRD patients.

#### Hepatitis A and B Vaccine

##### Efficacy–Immunogenicity–Safety

Since the 2011 recommendations, 11 cohort studies including in total 618 pedAIIRD patients and 278 healthy controls assessed the HAV or HBV vaccination, with a maximum level of evidence of 2B for immunogenicity and safety (18–22, 24–29).

Efficacy was not evaluated; all studies assessed immunogenicity. There is a good correlation between antibody concentration and level of protection against infection (65). In comparison to healthy controls, seroprotection rates were equal (19, 24) or reduced (26, 29), with lower antibody concentrations found in JIA and jSLE patients (19, 22, 24, 29). With regard to safety, no serious adverse events were reported and no effect on JIA and jSLE disease activity was observed (24, 29). Three of 28 periodic fever adenitis pharyngitis aphtosis (PFAPA) patients vaccinated with the HAV vaccine had a flare post-vaccination (20). No non-vaccinated PFAPA control group was available.

##### Influence of Treatment

The lower antibody concentrations found in JIA and sJLE patients were independent of medication use, including TNFα-inhibitors, but patient numbers were small (28, 29).

Based on these data, the NIP can be followed for pedAIIRD patients.

#### Meningococcal Vaccine

One new study was available that assessed the antibody persistence over time after vaccination against meningococcal type C (30). The seroprotection rates 4 years after vaccination were similar in 127 JIA patients compared to 1527 juvenile controls, with a trend toward faster waning of immunity in patients on bDMARDS (*n* = 7). Efficacy and safety were not assessed.

Based on these data, no specific recommendations were formulated for meningococcal vaccination, and the NIP can be followed.

#### Human Papilloma Virus Vaccine

##### Efficacy–Immunogenicity–Safety

Since 2011, 4 cohort studies were performed including 109 patients (89 JIA, 14 jSLE, 6 JDM) and 125 healthy controls that assessed the HPV vaccine (15–17, 50). Efficacy was not assessed, all studies evaluated antibody titers as surrogate endpoint. Although the HPV vaccine is 98–100% effective against cervical intraepithelial neoplasia (CIN) caused by HPV16/18 in healthy women (66), the exact correlation between antibody levels and protection against cervical carcinoma is unknown.

Seropositivity rates were generally similar in patients and controls. Antibody concentrations were similar in 68 JIA patients including 9 on TNFα-inhibitors compared to controls after the third vaccination in one study (15). However, lower antibody concentrations were found in another study with 21 JIA patients including 6 on TNFα-inhibitors, in 6 jSLE patients and 6 JDM patients (17, 50).

Regarding safety, adverse events were similar in patients and healthy controls. JIA disease activity was similar before and after vaccination. In total 10 SLE flares were described but no conclusions can be drawn as this included the adult population and no unvaccinated control group was available (16).

##### Influence of Treatment

Groups were too small for definite conclusions, no differences were described in antibody concentration or JIA disease activity in 6 JIA patients on TNFα-inhibitors compared to 15 JIA patients without TNFα-inhibitors (17).

Given the high risk of chronic HPV infection and HPV-associated carcinoma *in situ* in SLE patients (67), the high seropositivity rates after vaccination in patients with jSLE and other pedAIIRD, and the mild adverse events after vaccination, a specific recommendation was formulated for jSLE patients (3). For these patients in particular, the HPV vaccination should be strongly considered when jSLE patients have not (yet) been vaccinated.

#### Pneumococcal Vaccine (PCV10 PCV13 and PPSV23)

##### Efficacy–Immunogenicity–Safety

Six cohort studies assessed vaccination against pneumococci with either the PCV (10/13) or PPSV23 vaccine in pedAIIRD patients. These studies included 224 patients (56 jSLE, 27 JIA, 122 IBD, 19 CAPS patients), 56 healthy controls and 30 asthma patients as controls. None of the studies was powered to assess efficacy, but one patient on rituximab and one on a TNFα-inhibitor got pneumonia/pneumococcal invasive disease despite vaccination (31, 35). Regarding immunogenicity, the correlation between antibody levels and protection against infections has been previously shown in RA patients after the PCV7 vaccine (68). The humoral immunogenicity of the PCV10, PCV13 vaccine and PPSV23 vaccine was shown in patients with SLE (31, 36), IBD (32) and JIA (35), despite reduced antibody titers in some studies compared to controls.

Both the PCV10, PCV13 and PPSV23 vaccine were tolerated well without severe adverse events or disease flares (35, 36). In contrast, in CAPS patients, (severe) systemic reactions were described in 12 of the 15 PPSV23 vaccinations (34). This study included 1 pediatric patient receiving the PPSV23 vaccine; two pediatric CAPS patients receiving the PCV vaccine did not experience severe systemic reactions (33, 34).

##### Influence of Treatment

Rituximab reduced the seropositivity rates in 9 SLE patients (31), whereas antibody concentrations did not differ between patients (JIA *n* = 17, IBD *n* = 12) with and without TNFα-inhibitors (32, 35). The effect of csDMARDs was not assessed.

Based on the fact that pneumococcal conjugate vaccine is included in the NIP for all children, the high rates of immunogenicity among pedAIIRD patients and the favourable safety profile of the vaccine, the PCV10/13 vaccine is recommended for all non-vaccinated pedAIIRD patients.

#### Influenza Vaccine

##### Efficacy–Immunogenicity–Safety

Since 2011, 9 studies on the seasonal influenza vaccine and five on the pandemic H1N1 influenza strain vaccine were performed including 841 pedAIIRD patients and 457 healthy controls, although several patients seem to be included in more than one study considering the H1N1 vaccine (37, 39–49, 69).

Studies were underpowered to assess efficacy, but 3 studies described infection rates (42, 45, 46). In vaccinated patients, influenza like illness occurred less frequently than in unvaccinated patients whereas influenza rates were similar in vaccinated patients and vaccinated healthy controls (42, 46). Most studies assess immunogenicity, mainly defined as a protective level of antibodies measured by the haemagglutination inhibition assay. Most of the studies demonstrated similar high rates of immunogenicity among pedAIIRD patients and healthy controls after the seasonal influenza vaccine (41, 42, 45–49) and the H1N1 vaccine (37, 40). Two studies in a similar cohort of 118 jSLE patients showed reduced seroprotection and seroconversion rates in jSLE patients compared to controls, especially in patients on high dose glucocorticosteroids (>20 mg/day) (38, 40).

With regard to safety, influenza vaccination did not influence disease activity of the underlying disease in the majority of studies with patients with JIA and jSLE (37, 39, 42, 45, 48, 49). One study included unvaccinated patients as a control cohort and reported disease worsening in 35% of the vaccinated JIA patients vs. 23% of the unvaccinated JIA patients, however it should be mentioned that the unvaccinated patients had lower baseline disease activity (46).

##### Influence of Treatment

No influence of methotrexate on influenza immunogenicity was found in two studies (37, 47). Controversial results were found on the effect of TNFα-inhibitors on immunogenicity of the influenza vaccines. No effect of the TNFα-inhibitors was found on immunogenicity in 59 patients in 3 prospective cohort studies, were as lower protection rates (37, 47, 49) whereas lower seroprotection rates in patients on TNFα-inhibitors and lower H1N1-specific antibodies in 30 JIA patients were found in two prospective cohorts (42, 48). High dose glucocorticosteroids (>20 mg/day) impaired the immunogenicity of the H1N1 vaccine (38). Data on other csDMARDS and bDMARDS were scarce. Unfortunately no studies are yet available on timing of immunosuppressive drugs in pedAIIRD and the influenza vaccine.

Based on data retrieved from adult AIIRD patients showing increased susceptibility for severe influenza infections in immunosuppressed AIIRD patients (3), the fact that seasonal influenza vaccination is not incorporated in the NIP, the high rates of immunogenicity among pedAIIRD patients and the favourable safety profile of the vaccines, the task force again concludes that non-live seasonal influenza vaccination should be strongly considered for pedAIIRD patients treated with glucocorticosteroids or DMARDS.

### Live-Attenuated Vaccines

#### Measles, Mumps, Rubella Virus Booster Vaccine

##### Efficacy–Immunogenicity–Safety

Twelve studies were performed since 2011 including 1433 pedAIIRD patients that received the MMR or MMR/V (varicella) booster vaccine (12–14, 51–59). None of the studies evaluated efficacy, but there is a high correlation between antibody levels and protection against infection (65).

Immunogenicity of the MMR vaccine was similar to reduced compared to healthy controls (13, 51, 52, 57). During long-term follow-up (>1 year), several studies showed reduced seroprotection rates or antibody concentrations toward mumps (14, 53), measles (54) or rubella (14, 70).

Regarding safety, JIA disease activity was similar in patients randomized to be vaccinated compared to JIA controls (51). The MMR vaccine is a live-attenuated virus vaccine, we therefore focussed on vaccine-induced infections with the attenuated virus. No MMR-induced infections were detected after the booster MMR vaccine (58). This also included 132 patients on bDMARDs (51, 56, 58, 59).

##### Influence of Treatment

No effect of methotrexate or bDMARDS on immunogenicity of the MMR vaccine and on waning of MMR-specific antibody concentrations was detected in 132 patients using bDMARDS [TNFα-inhibitors (*n* = 123), anti-IL1 (*n* = 26) and anti-IL6 (*n* = 6)] (13, 14, 51, 57). The MMR (booster) vaccine did not induce severe adverse events or vaccine-induced infections. These data are of major importance in the new EULAR recommendations stating that the MMR booster can be considered in patients treated with bDMARDS, with most evidence currently available for TNFα-inhibitors. No data are available for the primary MMR vaccine as children are vaccinated shortly after the age of 1 year and pedAIIRD patients in these age groups are rare.

In addition, the lack of severe adverse events in pedAIIRD patients using methotrexate and the high levels of seroprotection in these patients have led to the recommendation that the MMR booster vaccination can be administered to patients using methotrexate.

#### Varicella Zoster Virus Vaccine

##### Efficacy–Immunogenicity–Safety

Five additional studies were available on the varicella zoster virus vaccine including 137 patients with pedAIIRD and 46 healthy controls, of which four studies evaluated primo varicella vaccination in naïve patients (59–63). Twenty-five patients from one study had been previously described in a study by Pileggi et al. (71). Additionally, 3 studies evaluated the MMR/V vaccine in 602 patients with pedAIIRD (56, 57, 72). For clarity, we distinguish between the studies on primo varicella vaccination in VZV naïve patents and the varicella booster vaccine.

##### Varicella Booster Vaccine

One study was a randomized controlled trial in which efficacy (herpes zoster infections) and safety (disease activity) was compared between 28 vaccinated jSLE patients and 26 unvaccinated jSLE patients after one booster VZV vaccination (level of evidence 1B). This study also included 28 age matched healthy controls who also received VZV vaccination consisting of ≥1,000 plaque-forming units of virus/0.5 mL (61). Immunogenicity: all patients had protective VZV antibodies pre-vaccination and after the booster VZV vaccination (61).

Regarding efficacy, the study of Barbosa on the VZV booster vaccination showed 4 herpes zoster cases in unvaccinated jSLE patients compared to no cases in vaccinated patients and controls. Both vaccinated SLE patients and controls had a significant increase in antibody levels between days 0 and 30. Regarding safety: The frequency of flares and the SLEDAI score were similar among the vaccinated and unvaccinated patients. None of the vaccinated patients experienced disseminated varicella rash or herpes zoster.

##### Varicella Primo Vaccination

Efficacy: Regarding the occurrence of varicella after primo vaccination, 3 varicella cases were described in uncontrolled studies in low responders whereas in another study no varicella infections were described during 3 years follow-up (60, 62, 63). Overall, studies were underpowered and not designed to assess efficacy.

Immunogenicity data showed a similar (increase in) VZV-specific geometric mean titers (GMTs) in patients and controls (60, 61). A seroconversion rate of approximately 80% was described after the primo vaccination (including patients who received both 1 and 2 vaccinations (57, 62). Patients who received two vaccines had significantly higher antibody concentrations than patients (*p* = 0.016) and HC (*p* < 0.001) who received only one vaccine (60). Antibody levels decreased over time but waning was not compared to healthy controls (57, 62).

Regarding safety of the VZV vaccine, primo vaccination induced varicella infections were described in 3 patients (1 using leflunomide, 1 using cyclosporine, 1 without medications) with self-limiting vesicular rash and in 1 sJIA patient using multiple immunosuppressive drugs (methotrexate, thalidomide, leflunomide, anakinra) who developed generalized vesicles after the booster vaccination and was admitted to the hospital and treated with acyclovir (58, 59, 62, 63). Ten other patients on anti-IL1 therapy or anti-IL6 therapy did not develop VZV-vaccine induced infections (59, 62, 63). Disease activity was similar in vaccinated patients and unvaccinated jSLE patients. Most studies did not describe disease flares, except for disease flares in an uncontrolled case series after VZV vaccination in 3 MKD patients (59). Overall, these data suggest that the VZV vaccine is in general well tolerated, but one should remain vigilant for VZV-induced infection.

##### Influence of Treatment

The effect of immunosuppressive treatment on efficacy and immunogenicity of the VZV vaccine was not systematically assessed. One study showed no effect of TNFα-inhibitors on the persistence of antibody levels (57) and no differences were found in seroconversion rates between 9 patients on low dose MTX vs. 14 patients with higher degrees of immunosuppressive drugs (63).

Regarding safety, vaccine-induced VZV infections, although rare and mainly self-limiting, were described in 3 patients who predominantly used immunosuppressive drugs or bDMARDS.

The VZV vaccination is included in the NIP of several European countries, and the question whether pedAIIRD patients can be effectively and safely vaccinated often rises. Based on the evidence described above with humoral immune responses comparable to healthy controls and lack of complicated or disseminated vaccine- induced varicella infections after VZV primo vaccination, and the risk of severe disseminated VZV infection in immunosuppressed hosts, the task force concluded that the VZV should be strongly considered in all varicella naïve patients on MTX and can even be considered under specific conditions in patients receiving TNFα-inhibitors, anti-IL1 and anti-IL6 therapy.

#### BCG Vaccine

No studies were performed to assess the BCG vaccine in pedAIIRD patients. However, there was one case report that described a fatal case of disseminated BCG infection in a newborn whose mother used infliximab (64). This casualty led to the safety warning in the updated recommendations.

#### Yellow Fever Vaccine

No studies were available to assess the outcomes of vaccination against yellow fever in pedAIIRD patients; data on this vaccine had to be extrapolated from studies performed in adult patients (3). There is limited data and mostly on the yellow fever booster vaccine. PedAIIRD patients usually require a primary vaccination dose. As fatal outcomes of YFV vaccination has been described in adult RA and SLE patients, and because adverse events tend to be more severe in patients with chronic inflammatory diseases, the task force recommended to withhold this vaccine from immunosuppressed pedAIIRD patients (73–75).

#### Effect of Biologic DMARDS on Vaccination Outcome

Since 2011, 39 studies including 811 patients with pedAIIRD focussed on the effect of bDMARDS on the outcome of vaccination (Table 2). Efficacy data are too scarce and often uncontrolled for definite conclusions. Most data are available for TNFα-inhibitors with 28 studies including 647 patients. In patients using TNFα-inhibitors more influenza like illness occurred in unvaccinated patients vs. vaccinated patients. Another study described one patient with invasive pneumococcal infection despite vaccination (37). Overall, vaccinations are able to induce an adequate (seroprotective) humoral immune response for most of the vaccines studied. There is a trend toward lower antibody concentrations and accelerated waning of humoral immunity in the studies assessing long term immunity (30, 42, 47, 48).

**Table 2:** The effect of bDMARDs on the efficacy, immunogenicity and safety of vaccinations in pedAIIRD.

**Medication**	**Studies**	**Pts on Med**	**Efficacy**	**Immunogenicity**	**Safety**	**Eff**	**Imm**	**Saf**
Anti TNFα	28	647	More influenza illness in unvacc vs. vacc. pts (Carvalho). Pneumococcal invasive dis. in 1 pt with TNFi: serotype infection not reported (Aikawa)	Adequate immunogenicity Pertussis, HAV, MMR. Influenza: equal seroprotection TNFi vs. DMARDs but lower titers and more rapid decline (Dell Era, Woerner, Carvalho)	No increase in DA or AE with (non) live vaccins. Fatal case of diss. BCG infection after BCG vaccination in child of mother using TNFi (Cheent)	4	2b	2b
Abatacept	4	32	NA	100% SP tetanus, 90% diphtheria after 2 mths, no HC (Brunner)	SAE 4, AE 29 (all abat-, no vacc.-related)	NA	4	4
Canakinumab	4	33	NA	Antibody titres in 4 pts only with PPV23: protective (Jaeger)	Not specified for pediatric vs. adult patients; 0% AE after PCV, AE to PPSV23 were more severe (flares). No SAE after MMR(v) booster, 1 flare (Jevaratnam)	NA	NA	4
Tocilizumab	9	64	1 SoJIA patient with influenza	SP, SC & GMT for influenza similar in SoJIA and HC (Shinoki)	4 pts with mild AE, none in HC. 2x arthritis post-vaccin in SJIA pt on anti-IL6 (Shimizu) and diarrhea after polio vaccine (Jeyaratnam)	4	2b	4
Rituximab	2	11	1 pt on RTX vacc with PCV got pneumonia (Gorelik)	RTX within 6 months pre-vacc reduced SP for PCV13 (Gorelik)	NA	4	4	NA
Anakinra	7	24	NA	NA	No SAE or MMR infection caused by MMR(/V) vacc (Heijstek Uziel), 1 VZV infection after VZV booster (Jevaratnam)	NA	NA	4

*LoE, level of evidence; Eff, Efficacy; Imm, immunogenicity Saf, Safety; NA, not analyzed; SP, seroprotection; SC, seroconversion; GMT, geometric mean titer; HC, healthy controls; pts, patients; DTP, diphtheria tetanus pertussis; IBD, inflammatory bowel disease; TNFi, tumor necrosis factor inhibitor; AE, adverse event; SAE, severe adverse event; SO, systemic onset; JIA, juvenile idiopathic arthritis; jSLE, juvenile systemic lupus erythematosus; bDMARD, biological disease modifying anti-rheumatic drugs; ABT, abatacept; TT, tetanus toxoid; MV, measles vaccine; RTX, rituximab; vacc, vaccine; AB, antibody; HAV, hepatitis A virus; HBV, hepatitis B virus; ETN, etanercept; ADA, adalimumab; TCZ, tocilizumab; IFX, infliximab; MMR(/V), measles mumps rubella (/varicella); VZV, varicella zoster virus; ANR, Anakinra; CAM, canakinumab; CAPS, cryopyrin-associated periodic syndrome*.

Regarding safety, non-live vaccines are well tolerated without induction of disease flares. An increasing amount of pedAIIRD patients received a live-attenuated MMR booster vaccine without vaccine induced infection (51, 58). The VZV (booster and primo) vaccine was well tolerated and no VZV vaccine-induced infections were noted in all vaccinated pedAIIRD patients on TNF-blocking agents. In contrast, the BCG vaccination caused fatal disseminated BCG infections in a newborn whose mother used infliximab (64).

Overall these data indicate that, in patients using TNFα-inhibitors, vaccines are largely immunogenic and safe. Primary live-attenuated BCG and YFV vaccines remain contra-indicated, but accumulating evidence supports the safety of the MMR booster and VZV vaccination in pedAIIRD patients using TNFα-inhibitors.

The effect of IL-1 blocking agents (anakinra, canakinumab) on outcome of vaccination was studied in 11 studies with 57 pedAIIRD patients, mostly with sJIA or CAPS. Efficacy data were not available and immunogenicity was not studied systematically, the studies focussed on safety of vaccination. In 10 patients who received the MMR booster and the MMR/V vaccination 1 case of varicella zoster was reported, but there were no severe vaccination-induced MMR infections (59, 72). In patients with periodic fever syndromes on IL-1 blocking agents, vaccinations (tetanus, influenza, PCV) were well tolerated, accept for the PPSV23 vaccine that caused severe adverse events in patients with CAPS. However, these data were also based on adult patients (34). Hence, we conclude that non-live vaccines are considered safe in pedAIIRD patients receiving IL-1 blocking agents, and that the live-attenuated MMR booster or varicella vaccines can be considered for these patients on a case-by-case basis.

In total 64 patients receiving vaccinations whilst treated with anti-IL-6 were described (Table 2). Patients with sJIA treated with anti-IL-6 inhibitors showed equal seroprotection rates and antibody responses after influenza vaccination compared to healthy controls (45). Patients on IL-6 who received live-attenuated booster vaccines also had no severe adverse events (58). In addition, 32 patients on abatacept were described. They had adequate seroprotection rates after DTP vaccination and no vaccine related adverse events (11).

B-cell depleting agents are mainly studied in adult AIIRD patients and per study in pedAIIRD patients the number of patients on B-cell depleting agents is low (3). In the study of Gorelik et al. 9 patients on rituximab showed reduced seropositivity rates after PCV vaccination compared to patients without rituximab (31). These data are in line with the data in adult patients (76). Since there is no evidence on efficacy or immunogenicity of tetanus vaccination in patients receiving B-cell depleting agents in the preceding 6 months, it is the experts' opinion that passive immunization with tetanus immunoglobulins should be considered in case of an event with high risk for a tetanus infection.

#### Discussion

In this systematic literature review 57 new studies on the safety, immunogenicity, and efficacy of vaccinations in pedAIIRD were critically appraised and summarized to serve as the basis for the updated 2021 EULAR vaccination recommendations for pedAIIRD. For the 2011 recommendations, only 27 studies were available (5). Three major outcomes were evaluated: efficacy (the capacity to prevent infection), immunogenicity (the capacity to induce immune responses) and safety (defined as severe adverse events and effect on disease activity). On the one hand, the outcomes of vaccinations included in the NIPs should be assessed in the pedAIIRD population; on the other hand the need for additional booster vaccinations or additional other vaccinations should be evaluated.

The efficacy of vaccinations included in the NIPs has been shown for healthy children and adolescents. There were no studies that were powered to assess efficacy of vaccinations in pedAIIRD. These kind of studies are difficult to perform as they require large scale studies in pedAIIRD patients, especially since the risk of infection is low due to high herd immunity. For the influenza vaccine, not included in the NIP, data on influenza infection rates had to be extrapolated from adult AIIRD patients [which show an increased risk for (complicated) influenza infections] and efficacy data for influenza vaccination are lacking (77). Therefore, immunogenicity had to be assessed as a surrogate endpoint for efficacy.

The immunogenicity of vaccines depend on the type of vaccination, disease type and medication use. In general, vaccinations were immunogenic in pedAIIRD patients, including patients using (predominantly low dose) glucocorticosteroids, methotrexate or TNF-blocking agents. During long-term follow-up, humoral immunity may wane faster in patients depending on the pathogen. In patients on high dose immunosuppressive drugs, especially prednisolone and B cell depleting therapies, measuring antibody concentrations should be considered (38).

Regarding safety, vaccinations do not increase the disease activity. This was shown by two randomized controlled trials in JIA and jSLE patients (51, 61). Safety concerns remain based on case reports of CAPS flares after the PPSV23 vaccination, but controlled studies are lacking (34). Besides disease activity, safety is an important issue in patients on high dose immunosuppressive drugs or biologicals who require a live-attenuated vaccine. Evidence has accumulated since 2011 that the live-attenuated MMR booster vaccine and the VZV vaccine do not cause complicated or disseminated vaccine-induced infections in patients on methotrexate, TNF-blocking agents and small numbers of patients on anti-IL1 or anti-IL6 treatment (51, 58–61, 63). Varicella skin vesicles can occur after primo vaccination and should be monitored (58–60). In contrast, evidence has also accumulated on severe adverse events after the YFV vaccine (in adult patients) and the BCG vaccine, reinforcing the previous recommendation that these vaccines should be withheld in immunosuppressed patients (64, 73–75).

In conclusion, evidence has grown that non-live vaccines included in the NIPs are immunogenic and safe in pedAIIRD patients. Also, evidence show that the MMR booster is safe in patients on MTX and that the MMR booster and VZV primo vaccination can be considered in patients using MTX and even TNF-blocking agents and anti-IL1 and anti-IL6 treatment. In addition to the NIP, the seasonal influenza vaccine can be considered in immunocompromised pedAIIRD patients based on the favorable immunogenicity and safety data. The current SLR also shows that individualized vaccination strategies are necessary in immunocompromised pedAIIRD patients, that take into account the actual risk of infections, the long-term persistence of immunity after vaccination, the safety of vaccinations in relation to specific pedAIIRD and the influence of (new) treatments on vaccination outcome. The current recommendations based on this SLR may add to improved vaccination strategies in this vulnerable patient population.

## Data Availability Statement

The original contributions presented in the study are included in the article/Supplementary Material, further inquiries can be directed to the corresponding author/s.

## Author Contributions

MJ, MH, and CR performed the SLR. MJ and MH primarily wrote the manuscript. All authors were involved in the Delphi meeting and voting and reviewed the manuscript. All authors contributed to the article and approved the submitted version.

## Funding

This work was funded by European League against Rheumatism (EULAR).

## Conflict of Interest

The authors declare that the research was conducted in the absence of any commercial or financial relationships that could be construed as a potential conflict of interest.

## Publisher's Note

All claims expressed in this article are solely those of the authors and do not necessarily represent those of their affiliated organizations, or those of the publisher, the editors and the reviewers. Any product that may be evaluated in this article, or claim that may be made by its manufacturer, is not guaranteed or endorsed by the publisher.
