# Supplementary material for: Efficacy, Immunogenicity and Safety of Vaccination in Pediatric Patients With Autoimmune Inflammatory Rheumatic Diseases (pedAIIRD): A Systematic Literature Review for the 2021 Update of the EULAR/PRES Recommendations
Source: Front Pediatr. 2022 Jul 6;10:910026. doi: 10.3389/fped.2022.910026 (PMC9298835; doi:10.3389/fped.2022.910026)
Supplement: Supplementary file 1 [file Table_1.DOCX]

**PICO**

**What is the efficacy, immunogenicity and safety of vaccines available for paediatric patients with autoimmune inflammatory rheumatic diseases (AIIRDs) ?**

**Population: Juvenile or pediatric AIIRDs**

[*systemic lupus erythematosus, antiphospholipid syndrome, juvenile idiopathic arthritis, systemic sclerosis, sjogren syndrome, mixed connective tissue diseases, relapsing polychondritis, giant cell arteritis, polymyalgia rheumatica, Takayasu arteritis, ANCA associated vasculitis [microscopic polyangiitis, granulomatosis with polyangiitis, eosinophilic granulomatosis with polyangiitis, polyarteritis nodosa, Behcet’s disease, anti GBM antibody disease, cryoglobulinemic syndrome, polymyositis, dermatomyositis, clinically amyotrophic dermatomyositis, inclusion body myositis, antisynthetase syndrome, eosinophilic myositis, ankylosing spondylitis, spondyloarthritis, psoriatic arthritis, periodic fever syndromes [familial Mediterranean fever, TNF receptor associated syndrome (TRAPS), cryopyrin associated syndrome (CAPS)]*

**Intervention: Immunization /vaccination**

[*Influenza, tetanus, diphtheria, pertussis, measles, mumps, rubella, varicella, herpes zoster, human papilloma virus, streptococcus pneumonia, hepatitis A, hepatitis B, Neisseria meningitides, hemophilus influenza, tick borne encephalitis, typhoid fever, yellow fever, BCG]*

**Comparison :** *Healthy controls, non- vaccinated*  *with AIIRDs, no controls*

**Outcomes : Efficacy (**Prevention of infectious disease **)**

***Immunogenicity (****seroprotection and or seroconversion****)***

***Safety*** (*effect on the underlying autoimmune disease or adverse effects from the vaccine)*

**In- and exclusion criteria**

Inclusion:

1. Patients with AIIRD < 18 years
2. Papers reporting at least one of the following outcomes of vaccination:
   1. Efficacy: incidence of vaccine preventable infections following vaccination
   2. Immunogenicity: seroprotection or seroconversion / fold increase / antibody concentrations / cellular immunity
   3. Safety
      1. Adverse events
      2. Effect of vaccination on disease activity
3. Study design: Randomized control studies, cohort studies (including data-base claims), cross-sectional studies, case control studies, case series >5
4. Papers published from September 2009 to Jan 2019

Exclusion:

1. non-rheumatic autoimmune diseases, malignancies, immunodeficiencies, transplantation, atopic diseases, animal studies, vaccine development, in vitro studies
2. non original papers, non-English papers, articles with no original data, abstract presented in scientific meetings, case reports
3. Papers included in the previous recommendations
4. Induction of AIIRD in previously healthy population

**Searches**

The following electronic databases will be searched: MEDLINE (Ovid), EMBASE (Ovid), and the Cochrane Library (Wiley). The reference lists of included studies will also be searched by hand.
