# Supplementary material for: Efficacy, Immunogenicity and Safety of Vaccination in Pediatric Patients With Autoimmune Inflammatory Rheumatic Diseases (pedAIIRD): A Systematic Literature Review for the 2021 Update of the EULAR/PRES Recommendations
Source: Front Pediatr. 2022 Jul 6;10:910026. doi: 10.3389/fped.2022.910026 (PMC9298835; doi:10.3389/fped.2022.910026)
Supplement: Supplementary file 2 [file Table_2.DOCX]

**Supplementary table 1. Oxford Centre for Evidence-based Medicine – Levels of Evidence**

| **Level** |  |
| --- | --- |
| 1a | Systematic review with homogeneity of randomized controlled trials |
| 1b | Individual randomized controlled trial (with narrow Confidence Interval) |
| 1c | ‘All or none’ |
| 2a | Systematic review with homogeneity of cohort studies |
| 2b | Individual cohort study (including low quality randomized controlled trials) |
| 2c | ‘Outcomes’ research, ecological studies |
| 3a | Systematic review with homogeneity of case-control studies |
| 3b | Individual case-Control study |
| 4 | Case series (and poor quality cohort and case-control studies) |
| 5 | Expert opinion without explicit critical appraisal |

**Supplementary table 2. Grades of recommendation**

| **Grade** |  |
| --- | --- |
| A | consistent level 1 studies |
| B | consistent level 2 or 3 studies ***or*** extrapolations from level 1 studies |
| C | level 4 studies ***or*** extrapolations from level 2 or 3 studies |
| D | level 5 evidence ***or*** troublingly inconsistent or inconclusive studies of any level |
